# NFV, an HIV-1 protease inhibitor, induces growth arrest, reduced Akt signalling, apoptosis and docetaxel sensitisation in NSCLC cell lines

**DOI:** 10.1038/sj.bjc.6603435

**Published:** 2006-11-28

**Authors:** Y Yang, T Ikezoe, C Nishioka, K Bandobashi, T Takeuchi, Y Adachi, M Kobayashi, S Takeuchi, H P Koeffler, H Taguchi

**Affiliations:** 1 Department of Hematology and Respiratory Medicine, Kochi University, Kochi Medical School, Nankoku, Kochi 783-8505, Japan; 2 Department of Tumor Pathology, Kochi University, Kochi Medical School, Nankoku, Kochi 783-8505, Japan; 3 Department of Internal Medicine, Kochi University, Kochi Medical School, Nankoku, Kochi 783-8505, Japan; 4 Division of Hematology/Oncology, Cedars-Sinai Research Institute, UCLA School of Medicine, Los Angeles, CA 90048, USA

**Keywords:** HIV-1 protease inhibitor, NSCLC, Akt, Bcl-2, GSK-3

## Abstract

HIV-1 protease inhibitor (PI), nelfinavir (NFV) induced growth arrest and apoptosis of NCI-H460 and -H520, A549, EBC-1 and ABC-1 non-small-cell lung cancer (NSCLC) cells in association with upregulation of p21^*waf1*^, p27 ^*kip1*^ and p53, and downregulation of Bcl-2 and matrix metalloproteinase (MMP)-2 proteins. We found that NFV blocked Akt signalling in these cells as measured by Akt kinase assay with glycogen synthase kinase-3*α*/*β* (GSK-3*α*/*β*) as a substrate. To explore the role of Akt signalling in NFV-mediated growth inhibition of NSCLC cells, we blocked this signal pathway by transfection of Akt small interfering RNA (siRNA) in these cells; transient transfection of Akt siRNA in NCI-H460 cells decreased the level of Bcl-2 protein and slowed their proliferation compared to the nonspecific siRNA-transfected cells. Conversely, forced-expression of Akt partially reversed NFV-mediated growth inhibition of these cells, suggesting that Akt may be a molecular target of NFV in NSCLC cells. Also, we found that inhibition of Akt signalling by NFV enhanced the ability of docetaxel to inhibit the growth of NCI-H460 and -H520 cells, as measured by MTT assay. Importantly, NFV slowed the proliferation and induced apoptosis of NCI-H460 cells present as tumour xenografts in nude mice without adverse systemic effects. Taken together, this family of compounds might be useful for the treatment of individuals with NSCLC.

Lung cancer is the leading cause of cancer death all over the world. Non-small-cell lung cancer (NSCLC) comprises adeno-, squamous and large cell carcinoma, and constitutes the majority of lung cancers. Despite the development of new treatment strategies using novel anticancer drugs, the 5-year survival rates have not improved dramatically (Nemunatis *et al*, 2000; Khuri *et al*, 2001). Therefore, novel approaches to the treatment of this disease are urgently needed.

The uncontrolled proliferation of cancer cells is often associated with alteration of growth factor receptors such as the epidermal growth factor receptor family in lung cancer. This is often accompanied with the activation of various intracellular signal pathways causing enhanced cellular proliferation and/or decreased apoptosis. Phosphatidylinositol 3-kinase (PI3K), which relays various signal pathways via the production of phosphatidylinositide lipids, has been identified as one of the core intracellular signalling molecules in the stimulation of growth factors, subsequently phosphorylating and activating a serine/threonine kinase, Akt/protein kinase B (PKB) (Ullich and Schlessinger, 1990; Franke *et al*, 1997; Toker and Cantley, 1997; Cantley and Neel, 1999). Akt phosphorylates several antiapoptotic proteins, including the Bcl-2 family member BAD (Datta *et al*, 1997), caspase-9 (Fujita *et al*, 1999; Di Cristofano and Pandolfi, 2000), the inhibitor of nuclear factor-*κ*B (NF-*κ*B), kinase IKK*α* (Di Cristofano and Pandolfi, 2000) and glycogen synthase kinase 3 (GSK-3) (Cross *et al*, 1995; Pap and Cooper, 1998), resulting in prolonged cell survival. Glycogen synthase kinase 3 was initially discovered as an enzyme that inactivates glycogen synthesis in response to insulin stimulation (Welsh and Proud, 1993). Recently, GSK-3 is considered to be involved in multiple cellular processes, such as metabolism (Cross *et al*, 1995), tumorigenesis (Rubinfeld *et al*, 1996), differentiation (Harwood *et al*, 1995; He *et al*, 1995) and apoptosis (Pap and Cooper, 1998). Activated GSK-3 suppresses cell proliferation and survival (Brunet *et al*, 1999; Kane *et al*, 1999). Several signal pathways, including Akt, control GSK-3 activity (Sutherland *et al*, 1993; Cohen and Frame, 2001). Akt phosphorylates GSK-3*α*/*β* on Ser^21^ and Ser^9^ to inactivate its kinase activity (Sutherland *et al*, 1993; Cross *et al*, 1995). Previous studies showed that the PI3K/Akt pathway is activated in a variety of cancers, including those from prostate, ovary, breast, pancreas and lung (Cheng *et al*, 1992; Izuishi *et al*, 2000; Brognard *et al*, 2001; Kulik *et al*, 2001). Thus, the PI3K/Akt/GSK-3 signal pathway might be a promising molecular target of cancer treatment.

Human immunodeficiency virus type 1 (HIV-1) protease inhibitors (PIs) have become important tools in the treatment of HIV infection; these include saquinavir mesylate (SAQ), ritonavir (RTV), nelfinavir mesylate (NFV) and indinavir sulphate (IDV). Recent studies showed that PIs possess antitumour activity, which is independent of their ability to inhibit HIV protease: we found that PIs induced growth arrest and differentiation of NB4 and HL-60 human myeloid leukemia cells, and enhanced the ability of all-*trans* retinoic acid (ATRA) to decrease proliferation and increase differentiation of these cells (Ikezoe *et al*, 2000). In addition, PIs induced growth arrest and apoptosis of multiple myeloma (MM) cells via inhibition of signalling through activated signal transducer and activator of transcription 3 (STAT 3) and extracellular signal-related kinase1/2 (ERK1/2) (Ikezoe *et al*, 2004b). More recently, we have shown that RTV blocked docetaxel-induced expression of *cytochrome P450 3A4 (CYP3A4)* and enhanced antitumour effects of docetaxel against DU145 human prostate cancer cells *in vitro* and *in vivo* (Ikezoe *et al*, 2004a). Moreover, other investigators showed that PIs can decrease proliferation of Kaposi sarcoma as well as prostate cancer cells via inhibition of NF-*κ*B activity (Pajonk *et al*, 2002; Pati *et al*, 2002; Sgadari *et al*, 2002).

In this study, we found that NFV blocked Akt signalling and induced growth arrest and apoptosis of human NCI-H460 and -H520 NSCLC cells *in vitro* and *in vivo*.

## MATERIALS AND METHODS

### Cell lines and cell culture

NCI-H520 (squamous cell carcinoma) and -H460 (large cell), A549 (adenocarcinoma), ABC-1 (adenocarcinoma) and EBC-1 (squamous cell carcinoma) human NSCLC cells were maintained in RPMI 1640 with 10% FCS.

### Chemicals

Saquinavir mesylate (Roche, Branchburg, NJ, USA), RTV (Abbott Labs, North Chicago, IL, USA) and NFV (Japan Tobacco Specification, Tokyo, Japan) were dissolved in 50% dimethyl sulphoxide (DMSO; Burdick & Jackson, Muskegon, MI, USA) to a stock concentration of 10^−2^ M and stored at −80°C. Docetaxel (Aventis Pharmaceuticals Inc., Tokyo, Japan) was dissolved in PBS to a stock concentration of 10^−4^ M and stored at 4°C. The PI3-kinase inhibitor LY294002 (Calbiochem, San Diego, CA, USA) was dissolved in DMSO at a stock concentration of 2 × 10^−2^ M and was used at 10–60 *μ*M. Insulin-like growth factor 1 (IGF-1) (Austral Biologicals, San Ramon, CA, USA) was stored at −20°C and was used at final concentration of 50 nM.

### MTT assay

NCI-H460 and -H520 cells (10^5^ ml^−1^) were incubated with various concentrations of either PIs (10–50 *μ*M) or LY294002 (10–60 *μ*M) for 3 days in 96-well plates (Flow Laboratories, Irvine, CA, USA). After culture, cell number and viability were evaluated by measuring the mitochondrial-dependent conversion of the tetrazolium salt, MTT (Sigma, St. Louis, MO, USA), to a coloured formazan product as previously described.

### Cell cycle analysis by flow cytometry

Cell cycle analysis was performed on NSCLC cells incubated for 48 h with NFV (20 or 30 *μ*M). The cells were fixed in chilled methanol overnight before staining with 50 *μ*g ml^−1^ propidium iodide in the presence of 1 mg ml^−1^ RNase (100 U ml^−1^; Sigma) and 0.1% NP40 (Sigma). Analysis was performed immediately after staining using a FACScan (Becton Dickinson, Mountain View, CA, USA).

### Assessment of apoptosis

Apoptotic cell death was examined by terminal deoxyribonucleotide transferase-mediated dUTP nick-end labelling (TUNEL) method. The TUNEL assay was performed using the *In Situ* Cell Death Detection kit (Roche Molecular Biochemicals, Mannheim, Germany), as previously described (Yang *et al*, 2004).

### Plasmid and transfection

NCI-H460 cells were transiently transfected with Gag-AKT expression vector (Burgering and Coffer, 1995) using the GenePORTER transfection reagent (Gene Therapy Systems, Inc., San Diego, CA, USA).

### Western blot analysis

NCI-H460 and -H520 cells (3 × 10^5^ ml^−1^) were plated in 60 mm plates and cultured with NFV (20–40 *μ*M, 24 h) or IGF-1 (50 ng ml^−1^, 30 min) either alone or in combination. After incubation, cells were washed twice in PBS, and whole-cell lysates were prepared as previously described (Yang *et al*, 2004). Proteins were resolved on a 4–15% SDS polyacrylamide gel, transferred to an immobilon polyvinylidene difluoride membrane (Amersham Corp., Arlington Heights, IL, USA), and probed sequentially with antibodies. Anti-p-Akt (Ser473) (Cell Signaling Technology, Beverly, MA, USA), -Akt (Cell Signaling Technology), p-ERK1/2 (Tyr202/Tyr204) (Cell Signaling Technology), ERK2 (sc-154, Santa Cruz, CA, USA), -p53 (Calbiochem, Darmstadt, Germany), -Bcl-2 (sc-7382, Santa Cruz), -Bcl-xL (Cell Signaling Technology), -matrix metalloproteinase (MMP)-2 (SC-10736), -p27 ^*kip1*^ (sc-527, Santa Cruz), -p21 ^*waf1*^ (Abcam Ltd, Cambridge, UK) and (-actin antibodies (sc-1615, Santa Cruz) were used. The blots were developed using the enhanced chemiluminescence kit (Amersham Corp.).

### Akt immunoprecipitation kinase assay

Serum-starved NCI-H460 cells (24 h) were cultured either with or without NFV (20 *μ*M). After 24 h, cells were exposed to IGF-1 (50 ng ml^−1^) for 30 min. Cells were harvested and cell lysates were prepared. Akt kinase assay was performed using Akt kinase assay kit (Cell Signaling Technology), according to the manufacturer's instructions. Briefly, 200 *μ*g of cell lysates were incubated for 12 h with protein G-agarose beads bearing anti-Akt on a rotator at 4°C to immunoprecipitate Akt. This precipitate was next used to phosphorylate a specific substrate, the recombinant GSK-3*α*/*β* protein expressed in *Escherichia coli*. Briefly, 1 *μ*g of recombinant GSK-3*α*/*β* was incubated with Akt-antibody–protein G–agarose complexes in the presence of magnesium/ATP mixture for 30 min at 37°C. Samples were boiled for 5 min, resolved on 10% SDS–PAGE, and transferred onto immobilon polyvinylidene difluoride membrane. The membranes were incubated sequentially with anti-p-GSK-3*α*/*β* (Ser^21/9^) and -Akt antibodies and the blots were developed using the enhanced chemiluminescence kit (Amersham Corp).

### Small interfering RNA (siRNA) transfection

Signalsilence Akt siRNA kit (Cell Signaling Technology) was utilised to downregulate Akt protein in NCI-H460 cells. In brief, NCI-H460 cells were transfected with siRNA (final concentration of 100 nM) using transfection reagent (Cell Signaling Technology). After 2 days, cells were harvested and subjected to Western blot analysis. The membrane was probed sequentially with anti-p-Akt, -Akt, -Bcl-2, -Bcl-xL and -*β*-actin antibodies. Control cells were transfected with nonspecific siRNA and cultured under the identical condition.

### Trypan blue exclusion test

Either nonspecific or Akt siRNA transiently transfected NCI-H460 cells were plated in 96-well plates (Flow Laboratories) and cultured for various durations as indicated. Cell number and viability were evaluated by Trypan blue exclusion test.

### Mice

A total of 10 male triple-deficient BALB/c nude mice at 6 weeks of age were purchased from JAPAN SLC. Inc. (Shizuoka, Japan), and were maintained in pathogen-free conditions with irradiated chow. Animals were bilaterally, subcutaneously (s.c.) injected with 2 × 10^6^ NCI-H460 cells/tumour in 0.1 ml Matrigel (Collaborative Biomedical Products, Bedford, MA, USA). Mice were divided randomly into two groups of five mice each. Either NFV (60 mg kg^−1^ mouse^−1^) or control diluent was administered orally five times a week. The treatment was begun 1 day after NCI-H460 cells were injected into mice and continued until mice were killed. The dose of NFV was determined by our preliminary studies (data not shown). Tumours were measured every week with vernier calipers. Tumour sizes were calculated by the formula: *a* × *b* × *c*, where ‘*a*’ is the length and ‘*b*’ is the width and ‘*c*’ is the height in millimeters. At the end of the experiment, animals were killed by CO_2_ asphyxiation and tumour weights were measured after their careful resection. Tumour tissue was collected for analysis. The experiments, approved by the Review Board of Kochi University, were performed in strict accordance with the UKCCCR guidelines (Workman *et al*, 1998).

### Terminal deoxyribonucleotide transferase-mediated dUTP nick-end labelling assay for tumour xenografts

Tumours were fixed for 12 h in 10% neutral-buffered formaldehyde after killing, tissue blocks were embedded in paraffin and stained with *In Situ* Cell Death Detection kit (Roche Molecular Biochemicals), and examined by microscope.

### Data analysis

Combination index (CI) of NFV and docetaxel in NSCLC cells was calculated using the median effect method of Chou and Talalay (1984) (Calcusyn Software available from Biosoft, Cambridge, United Kingdom). Combination index values less than 1 indicate synergy, a CI=1 indicates an additive effect and a CI more than 1 indicates antagonism between the two agents. The difference between two groups under multiple conditions was assessed by one-way analysis of variance (ANOVA) followed by Boneferroni's multiple comparison tests using PRISM statistical analysis software (GraphPad Software, San Diego, CA, USA). The non-parametric Mann–Whitney *U*-test was performed to assess the difference between two groups in the *in vivo* studies.

## RESULTS

### Effect of PIs on the proliferation and apoptosis of human NSCLC cells

**Figure 1:**
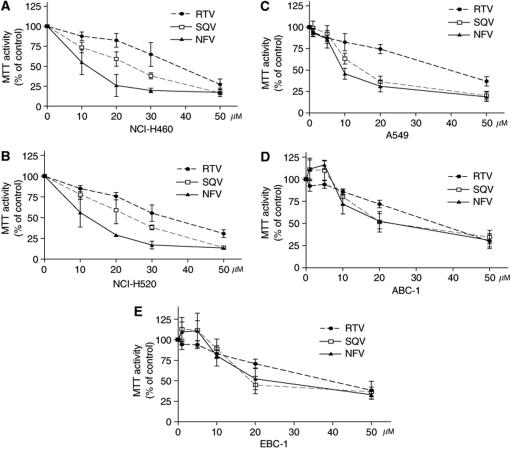
Effect of PIs on the proliferation and apoptosis of human NSCLC cells. Panels (**A**–**E**) MTT assay. NCI-H460 (**A**), NCI-H520 (**B**), A549 (**C**), ABC-1 (**D**), EBC-1 (**E**) cells (10^5^ ml^−1^) were plated in 96-well plates and cultured either with or without PIs (10–50 *μ*M). After 3 days, the cells were treated with MTT for 4 h at 37°C, and MTT activity was measured. Results represent the mean±s.d. of three experiments performed in triplicate.

The effect of PIs on proliferation of NSCLC cells was examined by MTT assay. Ritonavir, SAQ and NFV effectively inhibited the proliferation of both NCI-H460 (Figure 1A) and -H520 (Figure 1B) cells with an effective doses that inhibited 50% cell proliferation (ED_50s_) of approximately 40, 25 and 10 *μ*M, respectively, on the third day of culture. Similarly, the proliferation of A549 (Figure 1C), ABC-1 (Figure 1D) and EBC-1 (Figure 1E) was inhibited by PIs. Because NFV had the strongest antigrowth activity of the PIs, it was used in further analysis. We explored the effects of NFV on cell cycle distribution of NSCLC cells. NFV (20 or 30 *μ*M, 48 h) significantly increased the percentage of cells in the G_0_/G_1_ phase (Table 1). Concomitant with these changes, the percentage of cells in S phase significantly decreased (Table 1). Terminal deoxyribonucleotide transferase-mediated dUTP nick-end labelling assay was utilised to examine the proapoptotic effect of NFV. Nelfinavir induced apoptosis of these cells in a dose- and time-dependent manner (Table 2); NFV (20 or 40 *μ*M) induced a mean 19.3±4.4% (±s.d.) and 24.9±4.4% of the NCI-H460 cells to become apoptotic at 24 h. The TUNEL-positive population increased to a mean of 33.3±2.9 and 50.4±8.6% (*P*<0.005) at 48 h, respectively (Table 2). Similarly, NFV induced apoptosis of A549 cells in a dose- and time-dependent manner (Table 2).

### Nelfinavir modulates the levels of Bcl-2, p53, p21^*waf1*^, p27^*kip1*^ and MMP-2 in NSCLC cells

**Figure 2:**
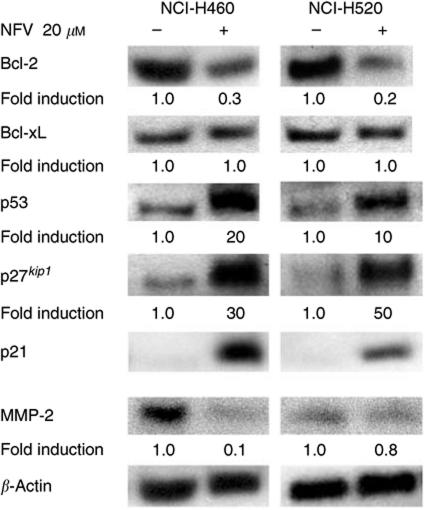
Nelfinavir modulates the levels of Bcl-2, p53, p21^*waf1*^, p27^*kip1*^ and MMP-2 in NSCLC cells. Western blot analysis. NCI-H460 and -H520 cells were cultured with either NFV (20 *μ*M) or control diluent. After 24 h, cells were harvested and subjected to Western blot analysis. The polyvinylidene difluoride membrane was sequentially probed with anti-p21^*waf1*^,-p27^*kip1*^, -p53, -Bcl-2, -Bcl-xL, -MMP-2 and -*β*-actin antibodies, and band intensities were measured by densitometry and normalised for *β*-actin. NFV, nelfinavir; RTV, ritonavir; SQV, saquinavir.

The effect of NFV on the expression of the antiapoptotic Bcl-2 family members was examined in NSCLC cells by Western blot analysis (Figure 2). Both NCI-H460 and -H520 cells expressed Bcl-2 and Bcl-xL proteins at a high level (Figure 2). Exposure of either NCI-H460 or -H520 cells to NFV (20 *μ*M, 24 h) decreased levels of Bcl-2 by either 70 or 80%, respectively (Figure 2). On the other hand, the levels of Bcl-xL were not modulated in these cells under the identical culture conditions (Figure 2).

We further explored whether NFV modulated the levels of the cell cycle checkpoint proteins, p53, p21^*waf1*^ and p27^*kip1*^ in NSCLC cells (Figure 2). NCI-H460 and -H520 cells possess the wild-type and mutant type of *p53* gene, respectively (Mitsudomi *et al*, 1992); exposure of these cells to NFV (20 *μ*M, 24 h) increased their levels of p53 by 20- and 10-fold, respectively, compared with control cells (Figure 2). Expression of p21^*waf1*^ was negligible in both cell lines; however, exposure of these cells to NFV dramatically induced expression of p21^*waf1*^ protein (Figure 2), suggesting that induction of p21^*waf1*^ mediated by NFV was p53-independent. Likewise, levels of p27^*kip1*^ were also markedly induced by NFV in NCI-H460 (30-fold) and -H520 (50-fold) cells compared with control cells (Figure 2).

The matrix metalloproteinases (MMPs) including MMP-2 degrade basement membranes and stromal extracellular matrix, resulting in tumour invasion and metastasis (Choi *et al*, 2005). NCI-H460 cells expressed MMP-2, and NFV downregulated this protein by 90% (Figure 2). NCI-H520 cells also expressed MMP-2, although the level was much lower than that in NCI-H460 cells. NFV slightly downregulated MMP-2 protein in these cells (Figure 2).

### Effect of NFV on Akt signalling in NSCLC cells

**Figure 3:**
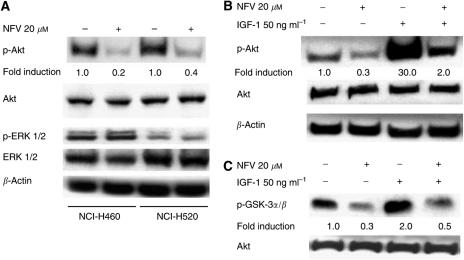
Effect of NFV on Akt signalling in NCI-H460 cells. Panels (**A**, **B**), Western blot analysis. (**A**) NCI-H460 and -H520 cells were cultured with either NFV (20 *μ*M) or control diluent. After 24 h, cells were harvested and subjected to Western blot analysis. The polyvinylidene difluoride membrane was sequentially probed with anti-p-Akt (Ser473), -Akt, p-ERK1/2 (Tyr202/Tyr204), -ERK1/2, and -*β*-actin antibodies, and band intensities were measured by densitometry. (**B**) NCI-H460 cells were cultured with either NFV (20 *μ*M) or control diluent. After 24 h, cells were exposed to IGF-1 (50 ng ml^−1^) for 30 min, harvested, subjected to Western blot analysis, and polyvinylidene difluoride membrane was sequentially probed with anti-p-Akt, -Akt, and -*β*-actin antibodies. Band intensities were measured by densitometry. (**C**) Akt kinase assay. NCI-H460 cells were cultured with either NFV (20 *μ*M) or control diluent. After 24 h, cells were exposed to IGF-1 (50 ng ml^−1^) for 30 min. Cells were harvested, proteins were prepared and subjected to Akt kinase assay with GSK-3 as a substrate. Membrane was sequentially probed with antibodies against -p-GSK3*α*/*β* (Ser ^21/9^) and –Akt. Band intensities were measured by densitometry. NFV, nelfinavir.

Both NCI-H460 and -H520 cells constitutively expressed the phosphorylated form of Akt (Figure 3A). Exposure of these cells to NFV (20 *μ*M, 24 h) decreased levels of p-Akt (Ser473) protein by 80 and 60%, respectively (Figure 3A). On the other hand, NFV (20 *μ*M, 24 h) was not able to downregulate levels of p-ERK1/2 (Tyr202/Tyr204) in these cells (Figure 3A). We further explored whether NFV downregulated IGF-1-stimulated expression of Akt in these cells. Exposure of NCI-H460 cells to IGF-1 (50 ng ml^−1^, 30 min) increased levels of the phosphorylated form of Akt by 30-fold. Preincubation of these cells with NFV (20 *μ*M, 24 h) decreased IGF-1-induced levels of the phosphorylated form of Akt by 80%. On the other hand, NFV did not affect levels of total Akt (Figure 3B). Furthermore, the effect of NFV on Akt signalling was investigated by the Akt kinase assay with GSK3*α*/*β* as a substrate (Figure 3C). NCI-H460 cells, which were serum-starved for 24 h, possessed measurable Akt activity (Figure 3C, lane 1). Similarly treated cells exposed to IGF-1 (50 ng ml^−1^, 30 min) increased the level of the phosphorylated form of the Akt substrate (GSK3*α*/*β*) by three-fold. Preincubation of these cells with NFV (20 *μ*M, 24 h) inhibited the IGF-1-stimulated Akt kinase activity by 73% (Figure 3C).

### Inhibition of Akt signalling by LY294002 enhanced the ability of NFV in NSCLC cells

**Figure 4:**
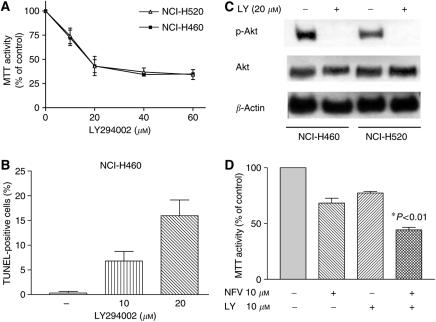
Effect of PI3 kinase inhibitor LY294002 on the growth of NSCLC cells. (**A**) MTT assay. NCI-H460 and NCI-H520 cells (10^5^ ml^−1^) were plated in 96-well plates and cultured either with or without LY294002 (10–60 *μ*M). After 3 days, the cells were cultured with MTT for 4 h at 37°C, and MTT activity was measured. Results represent the mean±s.d. of three experiments performed in triplicate. (**B**) Terminal deoxyribonucleotide transferase-mediated dUTP nick-end labelling assay. NCI-H460 cells were plated in 24-well plates and cultured either with or without LY294002 (10 or 20*μ*M); after 48 h, apoptosis was measured by TUNEL assay. Results represent the mean±s.d. of two experiments performed in triplicate. (**C**) Western blot analysis. NCI-H460 and -H520 cells were cultured with either LY294002 (20 *μ*M) or control diluent. After 24 h, cells were harvested, and subjected to Western blot analysis. The polyvinylidene difluoride membrane was sequentially probed with anti-p-Akt, -Akt and -*β*-actin antibodies. LY, LY294002. (**D**) LY294002 enhanced the ability of NFV in NCI-H460 cells. MTT assay. NCI-H460 cells (10^5^ ml^−1^) were plated in 96-well plates and cultured with NFV (10 *μ*M) or LY294002 (10 *μ*M) either alone or in combination. After 2 days, their viability was measured by MTT assay. The statistical significance was determined by ANOVA followed by Boneferroni's multiple comparison tests. Results represent the mean±s.d. of three experiments performed in triplicate.

To study the role of Akt signalling in survival of NSCLC cells, we blocked this pathway using a PI3 kinase inhibitor LY294002 (Vlahos *et al*, 1994). As shown in Figure 4A, LY294002 (20–60 *μ*M, 3 days) effectively inhibited the proliferation of both NCI-H460 and -H520 cells with an ED_50s_ of approximately 20 *μ*M. LY294002 (10 or 20 *μ*M, 48 h) induced apoptosis of NCI-H460 cells in a dose-dependent manner (Figure 4B). LY294002 (20 *μ*M, 24 h) completely blocked phosphorylation of Akt in these cells (Figure 4C). Furthermore, we explored the impact of blockade of Akt signalling by LY294002 on the effect of NFV in NSCLC cells; LY294002 significantly enhanced the ability of NFV to inhibit the growth of NCI-H460 cells (Figure 4D).

### Transfection of Akt siRNA in NCI-H460 cells

**Figure 5:**
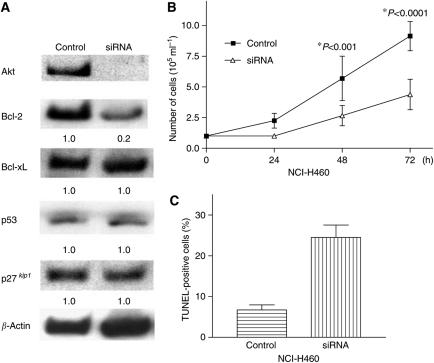
Transfection of Akt siRNA decreased levels of Akt and Bcl-2 and slowed the proliferation of NCI-H460 cells. (**A**) Transfection of Akt siRNA. NCI-H460 cells were transfected with either Akt siRNA or nonspecific siRNA. After 2 days, cells were harvested, proteins were extracted and subjected to Western blot analysis. Membrane was probed sequentially with antibodies against Akt, -Bcl-2, -Bcl-xL, -p53, p27^*kip1*^ and *β*-actin. Blots were developed using the enhanced chemiluminescence kit. (**B**) Trypan blue exclusion test. After 2 days from transfection, the control and Akt siRNA transiently transfected NCI-H460 cells were harvested, re-plated in 96-well plates, and incubated for 3 days. Cell numbers and viability were evaluated at the indicated time point by Trypan blue exclusion test. Results represent the mean±s.d. of three experiments performed in triplicate. (**C**) Terminal deoxyribonucleotide transferase-mediated dUTP nick-end labelling assay. NCI-H460 cells were transfected with either Akt siRNA or nonspecific siRNA. After 2 days, apoptosis was measured by TUNEL assay. Results represent the mean±s.d. of two experiments performed in triplicate. siRNA, small interfering RNA.

In order to block Akt signalling in NSCLC cells, double-stranded Akt siRNA molecules were delivered into the NCI-H460 cells. Transient transfection of these cells with Akt siRNA almost completely blocked expression of Akt protein (Figure 5A). Interestingly, the level of Bcl-2 protein decreased by about 80% in Akt siRNA-transfected cells, compared with nonspecific siRNA-transfected cells. In contrast, the levels of Bcl-xL, p53 and p27^*kip1*^ were not modulated after transfection of Akt siRNA (Figure 5A). The control and Akt siRNA transiently transfected NCI-H460 cells were incubated for 3 days in 96-well plates. The cell numbers and viability were evaluated by Trypan blue exclusion test on each day. The cell growth of Akt siRNA-transfected NCI-H460 cells was significantly slowed compared with the nonspecific siRNA-transfected control cells (*P*<0.001) (Figure 5B). Furthermore, we assessed whether downregulation of Akt by siRNA induced apoptosis of NCI-H460 cells. As shown in Figure 5C, 24±5% of Akt siRNA-transfected cells become apoptotic on the second day of culture (Figure 5C). On the other hand, merely 7% of control cells became apoptotic (Figure 5C).

### Forced-expression of Akt partially reversed NFV-mediated growth inhibition of NCI-H460 cells

**Figure 6:**
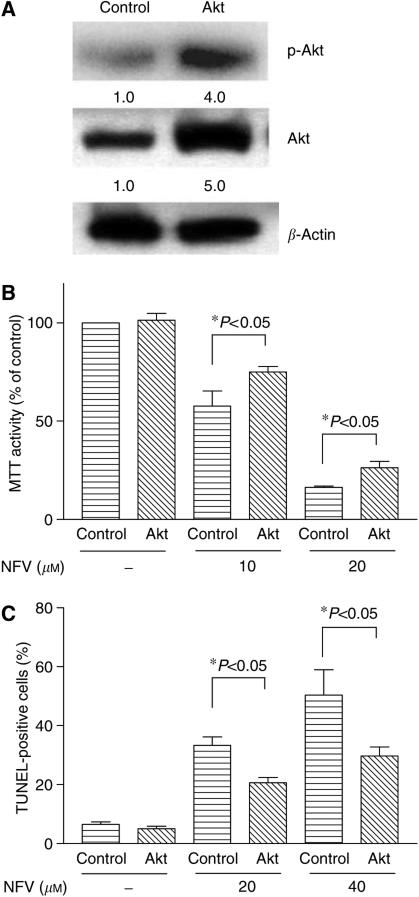
Forced-expression of Akt prevented NFV-mediated growth inhibition of NCI-H460 cells. (**A**) NCI-H460 cells were transiently transfected with either Akt or empty vector. After 48 h, cells were harvested and subjected to Western blot analysis to measure the level of p-Akt and total Akt. (**B**) At the same time, cells were exposed to either NFV (10 or 20 *μ*M) or control diluent for 48 h. At the end of the experiment, cell viability was measured by MTT assay. Results represent the mean±s.d. of triplicate wells. (**C**) At the same time, cells were plated in 24-well plates and cultured either with or without NFV (20 *μ*M and 40 *μ*M); after 48 h, apoptosis was measured by TUNEL assay. Results represent the mean±s.d. of two experiments performed in triplicate. NFV, nelfinavir.

NCI-H460 cells were transiently transfected with Akt expression vector or an empty vector. After 48 h of transfection, the levels of the phosphorylated and total Akt proteins were measured by Western blot analysis (Figure 6A). Also, cells were re-plated in 96-well plate and exposed to either NFV or control diluent for 48 h. Cell viability was measured by MTT assay (Figure 6B). Akt and empty vector transfected NCI-H460 cells possessed almost identical MTT activity (Figure 6B); however, when these cells were exposed to NFV (10 or 20 *μ*M, 48 h), MTT activity in Akt-transfected NCI-H460 cells was significantly higher than that in empty vector-transfected cells (Figure 6B). For example, NFV (10 *μ*M, 48 h) decreased MTT activity in empty vector-transfected cells by 47±2% compared to control cells. On the other hand, MTT activity in Akt-overexpressing cells cultured with 10 *μ*M NFV was inhibited by 39±4% (*P*<0.05). Next, TUNEL assay was utilised to examine the proapoptotic effect of NFV on Akt and empty vector-transfected NCI-H460 cells (Figure 6C). When these cells were exposed to NFV (20 or 40 *μ*M, 48 h), TUNEL-positive cells in empty vector-transfected NCI-H460 cells were significantly higher than that in Akt-transfected cells (Figure 6C). For example, NFV (20 *μ*M, 48 h) induced a mean 33.3±2.9% (±s.d.) of empty vector-transfected NCI-H460 cells to become apoptotic; on the other hand, only a mean 20.6±1.9% (±s.d.) of Akt-overexpressing cells become apoptotic (*P*<0.05). These results suggested that forced-expression of Akt partially reversed NFV-mediated growth inhibition of NCI-H460 cells.

### Inhibition of Akt signalling by NFV or LY294002 sensitised NSCLC cells to antiproliferative effect of docetaxel

**Figure 7:**
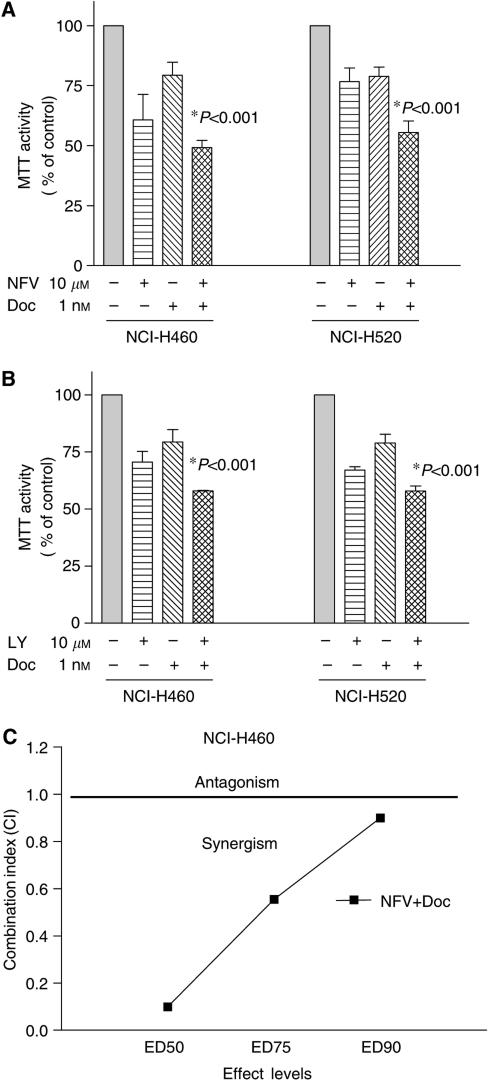
Nelfinavir (NFV) enhances antiproliferative effects of docetaxel in NSCLC cells. (**A**) NCI-H460 and -H520 cells (10^5^ ml^−1^) were plated in 96-well plates and cultured either with or without NFV (10 *μ*M). After 3 h, cells were exposed to docetaxel (1 nM) for 2 days, and their cells viability was measured by MTT assay. Results represent the mean±s.d. of three experiments performed in triplicate. (**B**) NCI-H460 and -520 cells (10^5^ ml^−1^) were plated in 96-well plates and cultured either with or without LY294002 (10 *μ*M). After 3 h, cells were exposed to docetaxel (1 nM) for 2 days and their viability was measured by MTT assay. The statistical significance was determined by ANOVA followed by Boneferroni's multiple comparison tests. Results represent the mean±s.d. of three experiments performed in triplicate. (**C**) NCI-H460 cells were cultured with NFV, docetaxel or both agents at various concentrations. Combination index (CI) was determined using the median effect method. A CI values less than 1 indicate synergy, CI=1 indicates an additive effect and a CI more than 1 indicates antagonism between the two agents. Doc, docetaxel; LY, LY294002.

We explored whether inhibition of Akt signalling by either NFV or LY294002 sensitised NSCLC cells to docetaxel-mediated growth inhibition. The NCI-H460 and -H520 cells were preincubated with NFV (10 *μ*M) for 3 h and exposed to docetaxel (1 nM). After 48 h, cell number was assessed by MTT assay (Figure 7A, left panel). Either NFV (10 *μ*M) or docetaxel (1 nM) alone inhibited the growth of NCI-H460 cells by 39 or 21%, respectively. The combination of NFV and docetaxel inhibited the growth of these cells by 51% compared to control cells (Figure 7A). Statistical analysis with ANOVA showed significant difference between either drug alone and combination of both (*P*<0.001). Similarly, NFV enhanced antiproliferative activity of docetaxel against NCI-H520 cells (Figure 7A, right panel). We next studied the interaction of LY294002 (PI3K inhibitor) and docetaxel (Figure 7B). Cells were preincubated with LY294002 (10 *μ*M) for 3 h and exposed to docetaxel (1 nM, 48 h). Either LY294002 (10 *μ*M) or docetaxel (1 nM) alone inhibited the growth of NCI-H460 cells by 30±4.0% or 21±5.0%, respectively. When cells were exposed to the combination of both, their cell growth was inhibited by 42±0.2% compared to control cells (*P*<0.001) (Figure 7B, left panel). The enhanced antiproliferative effect was also observed when NCI-H520 cells were treated in combination with LY294002 (10 *μ*M) and docetaxel (1 nM) (Figure 7B, right panel). We further analysed drug interaction employing median effect method. NCI-H460 cells were cultured with variety concentrations of NVF (1–40 *μ*M) or docetaxel (0.001–10 nM) either alone or in combination. Drug interaction analysis indicated that growth inhibition was synergistic (Figure 7C). The growth inhibition of NCI-H460 cells mediated by combination of NFV and LY294002 was also synergistic (data not shown).

### NFV induces growth arrest and apoptosis of NCI-H460 xenografts *in vivo*

**Figure 8:**
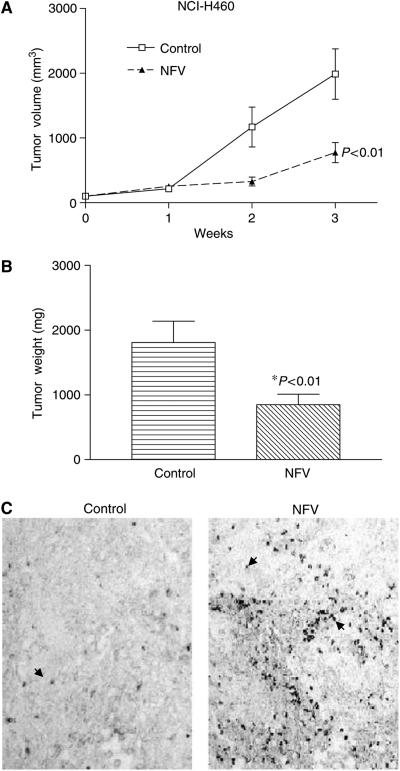
Effect of NFV in BALB/c triple-immunodeficient mice. (**A**) Tumour volumes. NCI-H460 cells were injected bilaterally s.c. into BALB/c nude mice, forming two tumours/mouse. Nelfinavir (60 mg kg^−1^ mouse^−1^) was administered orally five times a week. Control mice received diluent only. Tumours were measured every week. Each point represents the mean±s.e. of 10 tumours. (**B**) Tumour weights at autopsy. After 3 weeks of treatment, tumours were removed and weighed. Results represent mean±s.e. of tumour weights. Statistical significance of the differences was analysed by Mann–Whitney *U*-test. Bars, s.e.. (**C**) Terminal deoxyribonucleotide transferase-mediated dUTP nick-end labelling assay. NCI-H460 xenographs were dissected from the mice at the end of the experiment, fixed, and subjected to TUNEL assay to assess the proapoptotic effect of NFV *in vivo*. Arrowheads indicate representative TUNEL-positive cells (original × 200). NFV, nelfinavir.

We evaluated the ability of NFV to inhibit the growth of NCI-H460 cells growing as xenografts in triple-deficient murine model. Tumour volume was measured every week (Figure 8A), and tumour weights were determined at autopsy (Figure 8B). NFV markedly suppressed the growth and weights of NCI-H460 tumours. As shown in Figure 8A, the mean volume of NCI-H460 tumours in the mice who received NFV (774±465 mm^3^) was significantly decreased compared with control mice (2194±1041 mm^3^) (*P*<0.01). In addition, the difference of mean tumour weights between these two groups (849±514 mg NFV treated, 1809±1047 mg control diluent) was significant (*P*<0.01) (Figure 8B). No mouse showed signs of wasting or other signs of toxicity (data not shown).

In addition, we explored the ability of NFV to induce apoptosis of NCI-H460 cells *in vivo*. After 3 weeks of therapy with or without NFV, tumours were removed from nude mice and fixed. The induction of apoptosis was assessed by TUNEL assay. Apoptosis was induced in tumour cells from mice who received NFV. Conversely, apoptotic cells were barely detectable in control tumours (Figure 8C).

## DISCUSSION

HIV-1 protease inhibitor NFV inhibited Akt kinase activity, downregulated levels of Bcl-2, and induced growth arrest and apoptosis of NSCLC cells *in vitro* and *in vivo*. Growth inhibition mediated by NFV was p53-independent. Blockade of Akt signalling in NSCLC cells by transfection of Akt siRNA caused downregulation of Bcl-2 and proliferation was slowed (Figure 5). Phosphatidylinositol 3-kinase inhibitor LY294002 also decreased proliferation of NSCLC cells. Conversely, forced-expression of Akt in NSCLC cells partially reversed NFV-mediated growth inhibition of these cells. These results suggest that NFV inhibited the growth of NSCLC cells at least in part via inhibition of Akt signalling, with Bcl-2 playing a central role. These observations are reminiscent of studies done by other groups. Rapamycin, an inhibitor of mTOR downstream of Akt, reversed the neoplastic phenotype of prostate epithelial cells in transgenic mice expressing Akt (Majumder *et al*, 2004). When Bcl-2 was overexpressed in these mice, effect of rapamycin was reduced (Majumder *et al*, 2004), suggesting that Bcl-2 plays a pivotal role in apoptosis after the Akt/mTOR signalling pathway has been blocked.

Akt contributes to drug resistance of cancer cells. Recently, other investigators have shown that inhibition of Akt signalling sensitised cancer cells to chemotherapy; rapamycin overcame chemoresistance in murine lymphoma cells *in vivo* (Wendel *et al*, 2004). The PI3-K inhibitor LY294002 sensitised HL-60 human myeloid leukemia cells to doxorubicin-induced apoptosis (O’Gorman *et al*, 2000). In this study, we showed that NFV enhanced the ability of docetaxel to inhibit the growth of NSCLC cells (Figure 7B). Docetaxel has been used for individuals with NSCLC as a first-line therapy in combination with cisplatin; the effect of this combination therapy, however, is limited with a 1-year survival rate of less than 50% (Rigas, 2004). A clinical study using docetaxel and an Akt inhibitor is warranted.

This study found that NFV downregulated levels of MMP-2 and Bcl-2 in NSCLC cells (Figure 2). Recent studies showed that expression of MMP-2 was regulated by Bcl-2 at transcriptional and post-translational levels (choi *et al*, 2005). At the present time, it is unclear whether NFV downregulated the level of MMP-2 via inhibition of Bcl-2.

We previously showed that PIs induced growth arrest and apoptosis of MM cells in association with blockade of the STAT 3 and ERK1/2 signal pathways (Ikezoe *et al*, 2004b). We, therefore, investigated the effect of PIs on STAT 3 in NSCLC cells using an ELISA-based assay. Neither NCI-H460 nor -H520 cells possessed measurable STAT 3 DNA-binding activity; and AG490, an inhibitor of the Janus family of tyrosine kinases (JAK), upstream of STAT 3, was not able to affect their cell survival (data not shown). We also explore the effect of PIs on ERK signalling in these cells by Western blot analysis. Although NCI-H460 and -H520 cells constitutively expressed p-ERK protein, NFV was not able to downregulate levels of this cell survival factor in these cells (Figure 3A), suggesting that growth inhibition mediated by PIs is probably independent of inhibition of STAT 3 and ERK1/2 signalling in NSCLC cells.

Ritonavir and SAQ induced apoptosis of Kaposi sarcoma and prostate cancer cells via inhibition of NF-*κ*B activity (Pati *et al*, 2002; Ikezoe *et al*, 2004a). Nuclear factor-*κ*B is active and plays an important role in the development and progression of NSCLC (Tabata *et al*, 2001; Mayo *et al*, 2003). As we previously described, NCI-H460 and -H520 cells possess strong NF-*κ*B DNA-binding activity (Yang *et al*, 2004). Ritonavir inhibited NF-*κ*B DNA-binding activity in these cells by approximately 50% as measured by an ELISA-based assay (figure not shown); however, NFV was not able to block DNA-binding activity of this transcription factor (data not shown). At present, the difference of biological function between each PI remains unknown.

Insulin resistance has been identified in HIV-1-positive patients treated with NFV (Grinspoon, 2001). Insulin activates the PI3-K/Akt signalling, which stimulates glucose transport in adipose and muscle cells (Grinspoon, 2001). Recent studies showed that NFV blocked insulin-induced translocation of Akt to plasma membrane, where PI3-K phosphorylates Akt, resulting in inactivation of Akt (Ben-Romano *et al*, 2004). It could be the same as NFV inactivates Akt in NSCLC cells.

Pharmacokinetic studies showed that *C*_max_ was 6.66±2.33 *μ*g ml^−1^ (equals to 10 *μ*M) at 4.5 h when NFV 1000 mg was given to healthy volunteers (*n*=6, Japan Tobacco Specification, Tokyo, Japan). This concentration of NFV was able to inhibit growth of NCI-H460, -H520 and A549 cells in this study (Figure 1).

In summary, Akt signalling may be a promising molecular target for treatment of NSCLC. PIs might be useful as adjunctive therapeutic agents for the treatment of individuals with NSCLC and other types of cancer in which Akt signalling is active.

## Figures and Tables

**Table 1:** Effect of NFV on cell cycle distribution in NSCLC cells (%)

**NFV (*μ*M)**	**NCI-H460**	**NCI-H520**	**A549**	**EBC-1**
—	56.98±1.21	61.16±4.70	69.12±1.58	53.16±4.19
G0/G1 20	63.22±3.00	65.19±0.22	75.13±4.36	58.20±3.00
30	76.59±2.71**	78.33±3.07**	94.55±0.78**	83.35±1.63**
				
—	19.84±1.08	9.39±1.51	14.45±1.48	18.89±2.43
G2/M 20	11.57±3.52	8.33±1.07	10.90±1.27	13.71±5.13
30	11.15±4.59*	8.46±0.62	3.45±0.49**	7.00±0.14*
				
—	21.71±1.12	31.14±5.76	17.29±1.72	27.79±3.02
S 20	22.76±3.08	29.23±1.92	15.89±0.69	23.07±3.53
30	10.90±1.13**	13.11±3.27**	1.84±0.37**	8.05±0.39**

Cells were exposed to NFV (20 or 30 *μ*M). After 48 h, cell cycle distribution was analysed by FACscan. Significance of difference between control cells and cells cultured with NFV was calculated by paired *t*-test. Results represent the mean±s.d. of three experiments performed in triplicate.

* *P*<0.05;

** *P*<0.005. NFV=nelfinavir.

**Table 2:** Proapoptotic effect of NFV on NSCLC cells (TUNEL-positive cells, %)

	**Day 1**			**Day 2**		
**NFV(** *μ* **M)**	**—**	**20**	**40**	**—**	**20**	**40**
NCI-H460	1.0±0.3	19.3±4.4*	24.9±4.4*	2.4±0.3	33.3±2.9*	50.4±8.6*
A549	4.6±1.9	24.2±5.2*	36.6±2.1*	7.4±2.1	36.2±4.0*	59.1±7.2*

Cells were exposed to NFV (20 or 40 *μ*M). Apoptotic cells were measured by TUNEL assay on day 1 or day 2 of culture. Significance of difference between control cells and cells cultured with NFV was calculated by paired *t*-test. Results represent the mean±s.d. of two experiments performed in triplicate.

* *P*<0.01. NFV=nelfinavir; TUNEL=terminal deoxyribonucleotide transferase-mediated dUTP nick-end labelling.
